# Removal of Cr (VI) and microbial community analysis in PCB wastewater treatment based on the BESI^®^ process

**DOI:** 10.1371/journal.pone.0290023

**Published:** 2023-08-16

**Authors:** Jia Ouyang, Qinghua Miao, Dong Wei, Xinxin Zhang, Erming Luo, Chunying Li, Li Wei

**Affiliations:** 1 Guangzhou HKUST Fok Ying Tung Research Institute, Guangzhou, Guangdong, China; 2 School of Energy and Civil Engineering, Harbin University of Commerce, Harbin, China; 3 State Key Laboratory of Urban Water Resource and Environment, Harbin Institute of Technology, Harbin, Heilongjiang Province, China; 4 College of Life Sciences, Northeast Forestry University, Harbin, Heilongjiang, China; VIT University, INDIA

## Abstract

The treatment efficiency of Chromium (Cr)-containing Printed Circuit Board (PCB) wastewater is significantly hampered by the limited physiological activity of microorganisms when activated sludge is applied. In this study, the biodegradation and electron transfer based on sulfur metabolism in the integrated (BESI^®^) process use sulfur as the electron acceptor to achieve sulfate reduction and sulfide oxidation, leading to efficient removal of Cr. The concentrations of total Cr and Cr(VI) in the effluent were reduced to 0.5 mg/L and 0.1 mg/L, respectively, from an initial range of 25–32 mg/L in the influent. The removal of Cr (Δ*C*_(*Cr*(*VI*))_) mainly occurred in the Sulfate Reduction (SR) reactor, which was significantly correlated with the generation of sulphide (${\Delta C}_{{(S}^{2-})}$) (R^2^ = 0.9987). Meantime, analysis of the microbial community showed that Cr (VI) stress increased the diversity of the bacterial community in sludge. The presence of *Clostridium* (52.54% and 47.78%) in SR & Sulfide Oxidation (SO) reactor, along with the *Synergistaceae* (31.90%) and *Trichococcus* (26.59%) in aerobic reactor, might contribute to the gradient degradation of COD, resulting in a removal efficiency exceeding 80% when treating an influent with a concentration of 1000 mg/L. In addition, the main precipitation components in the SR reactor were identified by scanning electron microscope, indicating that Cr has been removed from wastewater as Cr(OH)_3_ precipitation. This study sheds light on the potential of using the BESI^®^ process for the real PCB wastewater treatment.

## Introduction

The composition of the discharged wastewater in Printed Circuit Board (PCB) industry is complex, mainly containing heavy metals such as chromium (Cr), various organic pollutants, acids and bases, and inorganic pollutants like NH_3_-N (NH_3_ or ammonium salt), phosphorus (P) (various phosphates). Cr, one of the toxic metals in PCB wastewater, poses a tremendous risk to human health and may even cause cancer [1]. Levels of Cr(VI) in groundwater, drinking water and sediments have surpassed the limits of 0.05 mg/L established in China and the control of chromium pollution in aquatic environments has attracted attention [2].

The removal technologies of Cr from wastewater included electrocoagulation, electrodeionization, electrochemical reduction, adsorption, biological treatment, membrane treatment and photocatalysis, etc. Biological treatment of Cr can convert the pollutants to a unique state without damaging the environment. Marine sulfate-reducing bacteria (SRB) can convert Cr(VI) into Cr(Ⅲ) by producing sulfide, which is the reason why Cr(VI) cannot be detected in the overlying water [3]. Somasundaram suggested that Cr ions can be synergistically removed from wastewater by SRB [4]. The removal efficiency of Cr(VI) exceeded 99% when the initial Cr concentration ranged from 22.7 to 74.9 mg/L in dye wastewater [5]. The combined effect of metals removal by anaerobic biomass under sulfate reducing conditions was found to have a high level of significance (P value < 0.05) on sulfate and COD removal [6]. Additionally, mesophilic SRB treating Cr-containing synthetic solution, achieved the removal of 82.1% Cr(VI), 76.9% sulfate and 85.7% COD [7].

Although current studies have shown that SRB are effective in removing Cr, few researchers have established a complete wastewater treatment process to investigate the effect of SRB on Cr-containing wastewater. So the experimental conclusions were difficult to guide the actual engineering application. Gong [8] put forward a mixotrophic denitrification system of biofilm-enhanced S^0^ recovery and Cr(VI) removal from composite wastewater, and suggested that the robustness of the reactor could be improved by bacteria enhancement. A bioreactor consisting of cellulose degradation-manganese (Mn) cycling bilayer carrier and two core strains was established to remove Cr ions from groundwater, achieving 95.37% Cr(VI) removal efficiency after 270 operating days [9]. In another investigation, a Sequencing Batch Reactor (SBR) for the microbial removal of Cr(VI) from wastewater with high chromium concentration (up to 1350 mg/L) was developed, leading to even 100% removal of Cr(VI) under anaerobic and mesophilic conditions for over 200 cycles [10].

However, we have previously found that Cr(VI) could be efficiently removed from synthetic water by optimizing the operating parameters of a single Up-flow Anaerobic Sludge Blanket (UASB) reactor. A community of mixed microbes may be more effective in removing Cr through biological synergy than a community consisting of sole SRB [11]. In this study, we attempted to remove Cr(VI) from Cr-containing PCB wastewater through a biodegradation and electron transfer based on sulfur metabolism integrated (BESI^®^) process, which was used for treating refractory industrial wastewater, such as nanofiltration concentrate and alkalinesurfactant-polymer (ASP) flooding [12, 13]. The effect of the BESI^®^ process on the removal of Cr and other pollutants from wastewater and the impact on the microbial community in each reactor were investigated. The removal mechanism and biological detoxification of Cr based on BESI^®^ process were further explored. This study provides theoretical guidance for the application of actual Cr-containing PCB wastewater treatment.

## Materials and methods

### PCB wastewater

The wastewater was raw PCB wastewater containing Cr, provided by Guangdong Huhui company. The influent Cr(VI) concentration was ranged from 25 mg/L to 32 mg/L. The pH was 2.0–3.0. The concentration of chemical oxygen demand (COD) was approximately 1000 mg/L.

### Experimental setup and operating conditions

**Fig 1:**
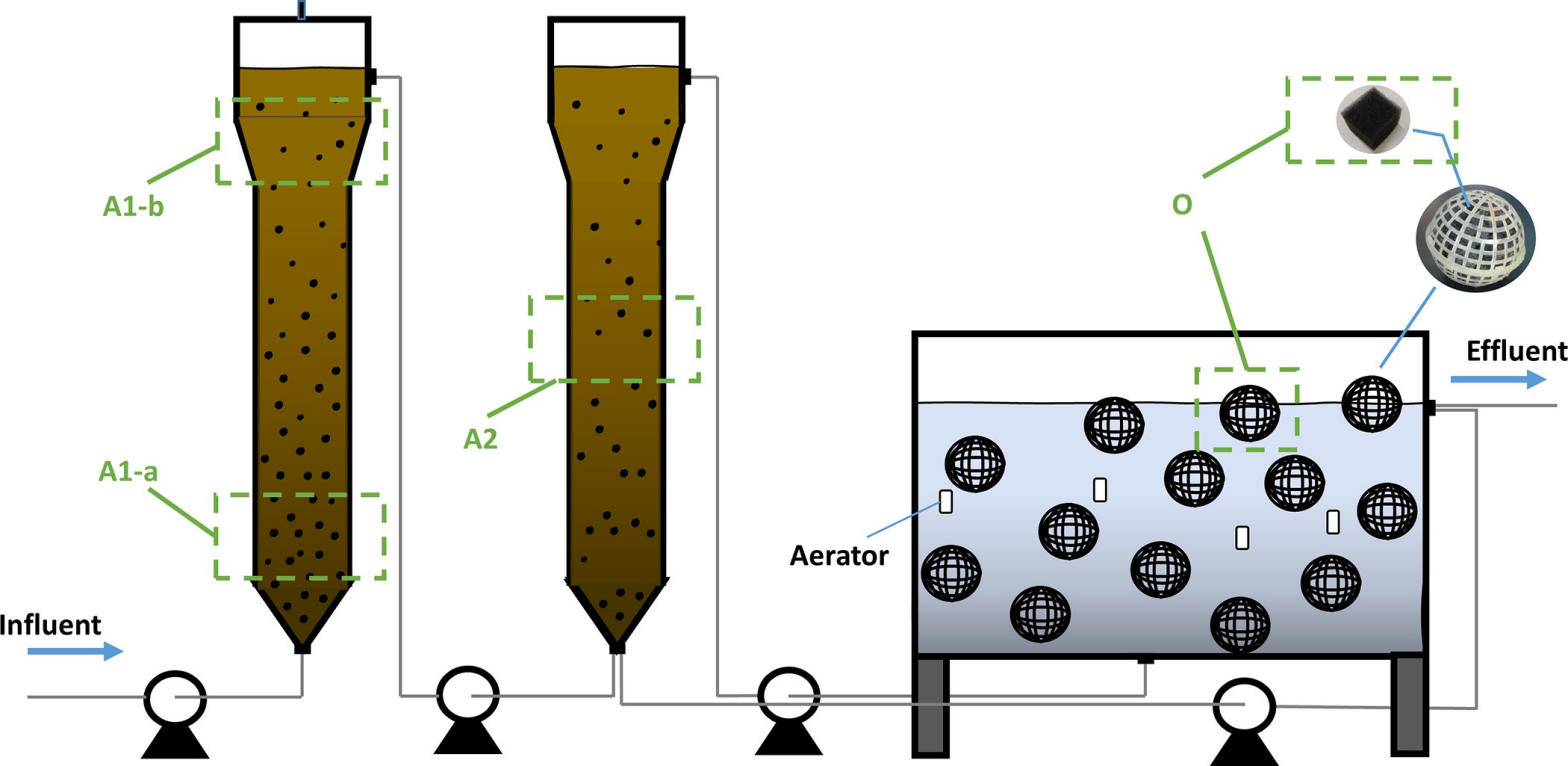
Schematic diagram of experimental setup.

The BESI^®^ process [12] was improved to enhance the treatment efficiency for Cr-containing wastewater (Fig 1). The system consisted of two UASB reactorsand one aerobic reactor in series. The first UASB was a sulfate reduction (SR) reactor in the anaerobic stage. The second UASB was the sulfide oxidation (SO) reactor, which was in the anoxic stage (the content of DO: 0.1–0.5 mg/L). The reactors were made of plexiglass to facilitate the observation of the growth and adhesion of sludge. The wastewater flowed through the three reactors in a bottom-up flow mode. The effective volume of the anaerobic reactor or the anoxic reactor was 50 L. To increase the sludge load of the bioreactor, approximately 50% of the reactor volume was filled with sludge. The effective volume of the aerobic reactor was 100 L. Black porous polyvinyl chloride cubes (packed in spherical plastic shells with a diameter of 0.1 m) were placed in the aerobic reactor (the DO content in this reactor was 3.0–4.0 mg/L), which increased the contact area of microorganisms and improved the removal ability of microorganisms to pollutants. The total filling rate was 40%, which provided enough space for the adhesion of sludge. The influent flow was 47 L/d. The hydraulic retention time (HRT) was approximately 3 days during the reactor start-up then it was adjusted to 24 h after 10 days.

The seeding sludge was provided by Guangzhou Nansha Sewage Treatment Plant. After being cultured, the sludge was seeded into the reactor. The SR and SO reactors were seeded with anaerobic sludge. The concentration of the sludge was approximately 4500–6000 mg/L. The sludge was acclimated using organic carbon and sulfate as substrates, which achieved high sulfate-reducing ability and denitrification ability. The aerobic process was primarily filler acclimation. One litre of sludge was seeded as the source of bacteria. New microbial films were generated on the surface of the fillers through bioaugmentation.

The operation of the reactor included the start-up stage (sludge acclimation stage) and the stable operation stage, with a total of 70 days of operation. Water samples were collected to analyse total Cr, Cr(VI), pH, sulfate and sulphide, COD and TOC.

### Analytical procedures

#### Physical and chemical analysis

Effluent samples from each reactor were collected every two days and measured instantly. The total Cr and Cr(VI) in water samples were determined by spectrophotometry with reference to GB7466-87. COD was determined by the dichromate method with reference to GB/T11914-89. TOC was detected using the TOC analysis reagent (Hach) by spectrophotometry. The pH value was measured with a multi-parameter water quality detector (Multi 3630 set F, WTW, Germany). Sulfide and sulfate (SO_4_^2-^) were detected using the sulphide and sulfate analysis reagent (Hach) by spectrophotometry.

At the end of the course (the 70th day), a sludge sample was taken from the anaerobic reactor. The sludge was placed in an oven at 105°C and dried to constant weight. Then the sludge cake was ground into powder. The elemental composition of the sludge was analysed by Scanning Electron Microscope (SEM) with Energy Dispersive Spectrometer (EDS) (JEOL-6700F SEM).

#### Microbial community analysis

After the reactor was stabilized, samples for microbial analysis were collected on the 65^th^ day: the activated sludge samples were collected from different positions of the anaerobic reactor and the anoxic reactor, and the biofilm samples was collected from the fillers in the aerobic reactor. The samples were briefly described in Table 1. The microbial community composition was analysed by high-throughput sequencing technology.

**Table 1:** Sludge samples.

Sample No.	Sample description
A1-a	Lower Anaerobic (bacteria)
A1-b	Upper Anaerobic (bacteria)
A2	Anoxic (bacteria)
O	Aerobic-black filler (bacteria)

## Results and discussion

### Reactor performance

#### Cr removal

**Fig 2:**
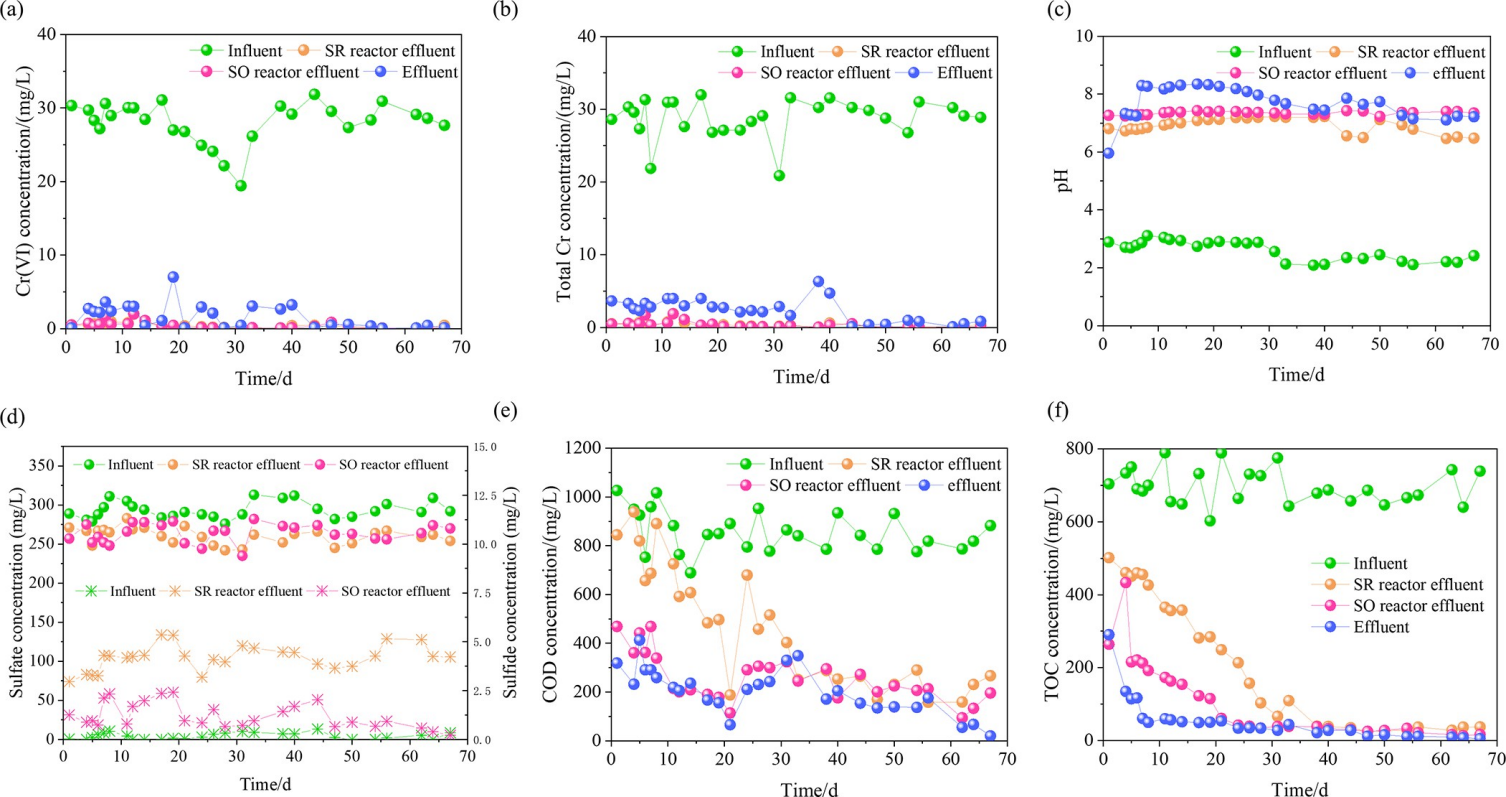
Effluent from the reactors. (a) Cr(VI) concentration; (b) total Cr concentration; (c) pH; (d) sulfate and sulphide; (e) COD concentration; and (f) TOC concentration.

The change in Cr(VI) concentration is shown in Fig 2(A). The influent Cr(VI) concentration was ranged from 25 mg/L to 32 mg/L. After the biological treatment process, the Cr(VI) concentration in the effluent of the SR reactor was reduced to below 0.1 mg/L, indicating a good removal efficiency. The Cr(VI) concentration in the effluent of the SO reactor was basically maintained below 0.1 mg/L except for fluctuations. The Cr(VI) concentration of aerobic effluent was high in the early stage of the operation, which might have been caused by the unstable treatment of wastewater in the early sludge acclimation stage. When the operation had stabilized, the Cr(VI) concentration of the aerobic effluent stayed below 0.1 mg/L. The Cr(VI) concentration in the effluent met the discharge limit requirements of the national standard “Discharge standard for pollutants from electroplating” (GB 21900–2008) and the Guangdong Provincial standard “Discharge standard for water pollutants from electroplating” (DB 44/1597-2015).

As shown in Fig 2(B), the change of total Cr was similar to Cr(VI). The SR reactor removed much of the total Cr. The total Cr concentration of effluent from the SR and SO reactors was basically below 0.5 mg/L except for fluctuations. The total Cr concentration of the aerobic effluent was relatively high at the beginning. After the operation had stabilized, the total Cr concentration of the aerobic effluent stayed under 0.5 mg/L, meeting the total Cr discharge standard.

#### pH value

The pH value of the effluent from each reactor is shown in Fig 2(C). As the influent pH was between 2.0 and 3.0, Cr ions were Cr_2_O_7_^2-^, HCrO_4_^-^ and Cr(Ⅲ) co-existing at this time [14–16], and mainly existed in the form of Cr(VI). So it showed that the total Cr concentration of the influent was approximately 30.88 mg/L, while the Cr(VI) concentration was approximately 24.48 mg/L. After anaerobic treatment, the pH of the effluent increased to 6.0–8.5, which had already reached the requirement of the discharge standard. Besides, the concentration of Cr was also dropped to nearly 0 mg/L. It indicates that the removal of Cr was correlated with the gain/loss of H^+^, which helped Cr(Ⅲ) to precipitate Cr(OH)_3_^0^. After the anoxic and aerobic treatments, the effluent pH fluctuated little.

#### Sulfate and sulfide

The changes in sulfate and sulfide during the anaerobic and anoxic stages are shown in Fig 2(D). The sulfate concentration in the influent was approximately 300 mg/L. Sulfate was consumed in the SR reactor while sulfide was produced, indicating that bacteria in the SR reactor metabolized sulfate into sulfide. During the entire operation, the average consumption of sulfate was approximately 32.96 mg/L, while the average concentration of sulfide in the anaerobic effluent was approximately 4.01 mg/L. It indicated that approximately 63.56% of sulfur was present in the sludge, which was lower than the 90–95% reported by Qian et al. [11] but higher than the 61.30% reported by Wei et al. [12]. This depended on the capacity of sulfate reduction and sulfide oxidation in the reactor. The more sulfate ions were reduced and utilized, the more elemental sulfur was oxidized in sludge.

#### COD and TOC removal

The change in COD concentration is shown in Fig 2(E). The COD in the influent fluctuated by approximately 1000 mg/L. In the anaerobic stage, the COD concentration of anaerobic effluent was high in the early sludge acclimation stage. When the operation had stabilized, the COD concentration of the anaerobic effluent was as low as 158 mg/L. As reported by Wei et al. [12], the utilization of sulfate contributes to the removal of COD. The theoretical ratio of sulfate to COD is 1:2, that is, for every 1 mole of sulfate consumed, approximately 2 moles of COD can be removed [12, 13, 17]. While in this study, the formation of sulfide was correlated with the removal of COD. In the early sludge acclimation stage, with the increase of sulfide production, the removal efficiency of COD is gradually significant. On the 20th day of operation, the COD concentration of the anaerobic reactor is the lowest (188 mg/L) while the sulfide production reaches the maximum (5.35 mg/L). However, the anoxic and aerobic stages removed COD better than the anaerobic stage, and the removal efficiency of COD reached 80%.

The change in TOC concentration was similar to COD. As shown in Fig 2(F), the anoxic and aerobic reactors had good TOC removal effects. Additionally, the removal of TOC significantly increased with the reactor running time prolonging.

### Evolution of pollutants removal

#### The interaction of Cr, pH, sulfate/sulphide and COD

The results of Fig 2(A) and 2(B) showed that the removal of Cr ions from wastewater mainly occurred in the SR reactor. The total Cr concentration was reduced from 29.42±2.00 mg/L (influent) to 0.36±0.18 mg/L (anaerobic effluent). Moreover, the changes in the concentration of sulfate, sulfide and pH were correlated with the removal of Cr(VI). Sulfate and H^+^ were consumed to form sulfides. Meanwhile, the production of sulfide promoted the reduction of Cr(VI) to Cr(Ⅲ). In the SO reactor, the sulfate concentration was higher than that in the SR reactor, while sulfide was lower. It means sulfide was converted into sulfate [13].

Besides, the isoelectric point of Sulfate Reducing Bacterium (SRB) is close to neutrality or acidic [18, 19]. When pH is above its isoelectric point, SRB is negatively charged and thereby has an electrostatic adsorption effect on Cr(VI). Additionally, the preferred pH range for SRB is 5.5 to 10 [20]. Cr(VI) adheres to the surfaces of microbial cells. Chemical bonds are formed between the functional groups on the cell surface and the metal particles. The adhesion of Cr(VI) to the surface of microbial cells, either converts Cr(VI) into Cr(Ⅲ), or accelerates the formation of Cr(Ⅲ) [21–23]. The conversion from Cr(VI) to Cr(Ⅲ) is either spontaneous or catalysed by chromate reductase [24]. So an increase in pH promotes the adsorption and removal of Cr(VI) by SRB. At the same time, some of the metabolites secreted by SRB can form metal complexes with Cr(VI), as they adhere to the cell membrane, thereby precipitating together with the bacteria.

As studied by Wei et al. [12], sulfate or other oxidized sulfides were as electron acceptors to dissimilate organics under anaerobic conditions. So the results of 3.4 contributed to the changes of sulfate and removal of Cr ions. Sulfate reacts with reducing substances (COD and TOC) to generate S^2-^, HS^-^, or molecular H_2_S, each of which is further converted into sulfur or thiosulfate. Then the generation of S^2-^, HS^-^, or molecular H_2_S promoted Cr(VI) converting into Cr(Ⅲ). Besides, the remaining COD and soluble sulfides are removed under aerobic conditions.

#### Relationship between Cr removal and sulfate/sulfide in the SR reactor

To further explore the relationship between Cr removal and other substances, the SR reactor was run again with influent COD concentrations of 200 mg/L (glucose as the carbon source), 300 mg/L of SO_4_^2-^ (anhydrous sodium sulfate as the SO_4_^2-^ source), control pH with 3.0–4.0, and a gradient of increasing total Cr concentrations (10 mg/L, 15 mg/L, 20 mg/L, 25 mg/L and 30 mg/L). The effluent concentration of Cr(VI), SO_4_^2-^, S^2-^ were tested (Table 2).

**Table 2:** Water quality of the SR reactor.

	Phase Ⅰ	Phase Ⅱ	Phase Ⅲ	Phase Ⅳ	Phase Ⅴ	Phase Ⅵ
Influent (Unit: mg/L)						
Total Cr	10.00	15.00	20.00	25.00	30.00	25.0
						(Refill)
Cr(VI)	9.10	11.39	17.60	18.35	27.28	18.35
SO_4_^2-^	301.00	304.00	294.00	277.00	297.00	-
S^2-^	0.28	ND	ND	0.04	0.01	-
Effluent (Unit: mg/L)						
Total Cr	0.29	1.03	0.50	0.36	-	-
Cr(VI)	0.14	0.08	0.05	0.01	0.02	1.62
SO_4_^2-^	212.00	290.00	288.00	285.00	243.00	-
S^2-^	0.84	1.10	2.75	3.07	1.87	0.07
Data Model						
△C_(*Cr*(*VI*))_	8.96	11.31	17.55	18.34	27.26	-
${△C}_{{({SO}_{4}}^{2-})}$	89.00	14.00	6.00	-8.00	54.00	
${△C}_{{(S}^{2-})}$	-0.56	-1.10	-2.75	-3.03	-1.86(eliminate)	
${△C}_{{(S}^{2-})}=0.2627{△C}_{\left(Cr(VI)\right)}+1.8289\;R^{2}=0.9987$						

Note: ND: not detected.

${△C}_{\left(Cr(VI)\right)}=C_{(Cr(VI))influent}-C_{(Cr(VI))effluent}$.

${△C}_{{({SO}_{4}}^{2-})}=C_{{({SO}_{4}}^{2-})influent}-C_{{({SO}_{4}}^{2-})effluent}$.

${△C}_{{(S}^{2-})}=C_{{(S}^{2-})influent}-C_{{(S}^{2-})effluent}$.

As shown in Table 2, in the influent, the average SO_4_^2-^ concentration was maintained at 294.60±7.28 mg/L, and almost no S^2-^ was detected. While the S^2-^ concentration increased from 0.28 mg/L to 0.84 mg/L with the SO_4_^2-^ concentration decreased in the influent Cr concentration of 10 mg/L. It indicated that anaerobic microorganisms in the reactor produced S^2-^ metabolites from SO_4_^2-^.

Moreover, as the Cr concentration in the influent increased from 10 mg/L to 25 mg/L, the cumulative concentration of the S^2-^ increased from 0.84 mg/L to 3.07 mg/L. Besides, the highest sulfate consumption (${△C}_{{({SO}_{4}}^{2-})}$) was appeared at the initial Cr concentration of 10 mg/L and at the highest Cr concentration of 30 mg/L. The value of ${△C}_{{({SO}_{4}}^{2-})}$ reached up to 89 mg/L, which may be the resistance of SRB to the impact of Cr. Because SRB can utilize sulfate occupying the channel for salt ions to enter the cells, competitively inhibiting the absorption of chromium ions by cells. Since chromate and sulfate have similar chemical structures (four tetrahedral oxygen atoms and two negative charges), Cr(VI) enters the cell mainly through sulfate channel proteins [25]. So it can resist the toxic effect from the influent Cr [26–28]. This is consistent with the results of Gu et al. [29] that a higher sulfate concentration led to less Cr(VI) entering the bacteria, a less toxic effect of Cr(VI) on the bacteria, a higher extracellular Cr(VI) concentration that the bacteria could tolerate, and a weaker adverse effect on the growth and reproduction of the bacteria.

However, there is a linear correlation between the removal of Cr (△C_(*Cr*(*VI*))_) and the production of sulphide ($‐{△C}_{{(S}^{2-})}$), and the fitting coefficient (R^2^) reached 0.9987. It indicated that the removal of Cr in SR reactor was significantly correlated with the conversion of sulfate/sulphide, which is a slight discrepancy with the results of Qian et al. [11]. It reported that the reduction of SO_4_^2-^ (biospecific SR rate) was inhibited by 21–47% when 50 mg/L of Cr(VI) was added to the reactor. But high concentrations of Cr(VI) inhibited the metabolic activity of microorganisms [30], 30mg/L of Cr(VI) in this study was the maximum.

### Microbial community analysis

#### Diversity of microorganisms in the BESI^®^ process

Table 3 shows the total number of sequencing reads available representing Operational Taxonomic Units (OTUs) in samples obtained from each reactor of the BESI^®^ process. The coverage rate for the samples (> 99.8%) indicated that a high proportion of the reads could be explained by the OTUs [31]. The most OTUs were found in anaerobic sludge (A1-a), which represented the inlet section of the reactor. The high diversity of the microbial community helped microorganisms resist the impact of wastewater from high concentrations of COD and toxic Cr ions. Along the direction of wastewater flow, the OTU value followed the order of A1-a>A1-b>A2>O; the diversity of the microbial community decreased as the pollutants were biodegraded and removed. Additionally, the lower Simpson indices (closer to 0, not closer to 1) in Table 3 also shows that the microbial samples were rich in diversity.

**Table 3:** Diversity indices of microorganisms in different biological phases.

Sample ID	Reads	97% Confidence interval					
		OTUs	ACE	Chao	Coverage	Shannon	Simpson
A1-a	29892	532	535	540	0.9995	4.79	0.0216
A1-b	27917	307	332	333	0.9984	2.57	0.2622
A2	32139	298	321	331	0.9986	2.41	0.2367
O	34091	267	284	293	0.9991	3.08	0.1286

#### OTU distribution and Venn distribution

**Fig 3:**
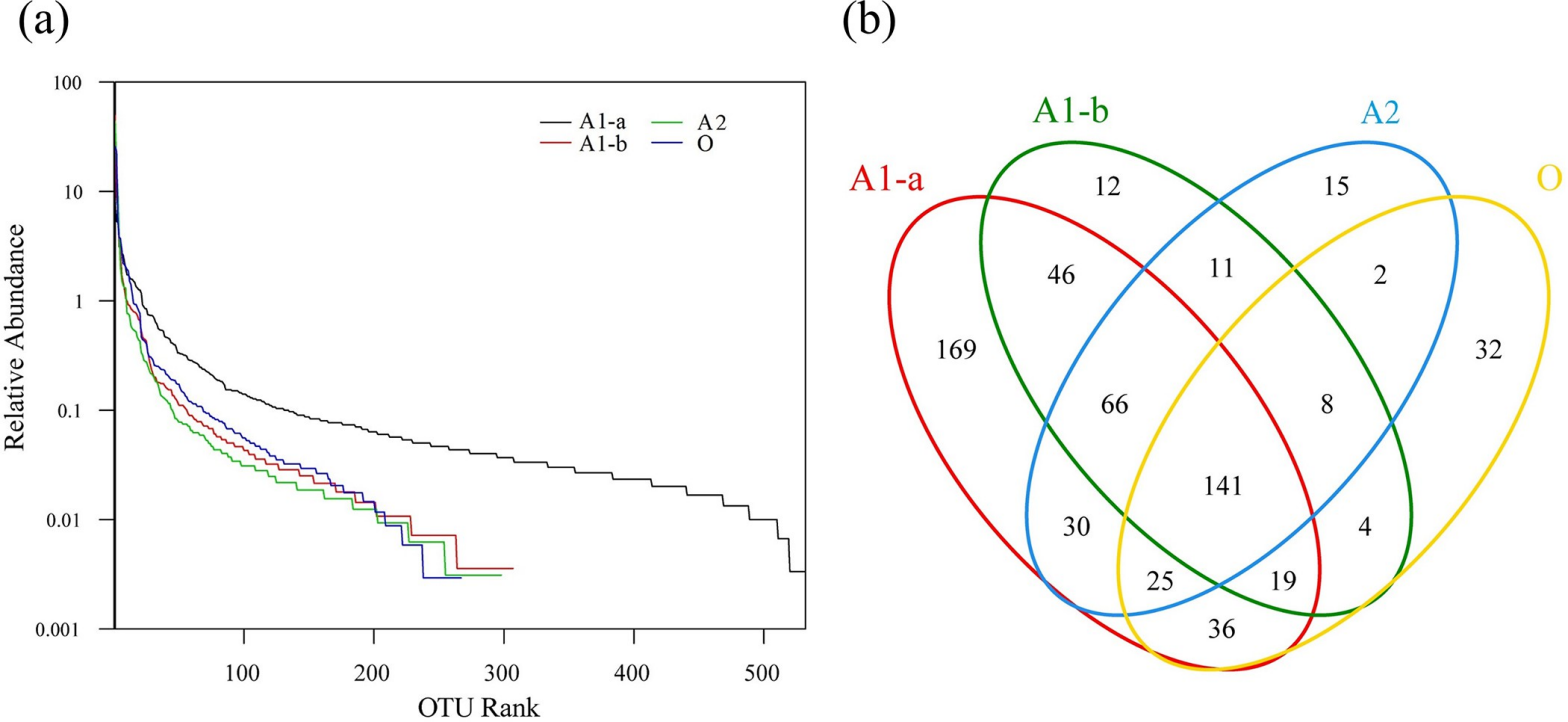
Composition of the microbial community in the reactor. (a) OTU distribution; (b) Venn distribution.

As shown in Fig 3(A), A1-a had a relatively flat OTU curve and the widest OTU distribution, indicating that A1-a had the highest microbial diversity, the conclusion of which was consistent with Table 3. However, the flatness of the OTU curves from other sludge samples was relatively poor, meaning that the evenness of the species distributions was low.

The Venn diagram (Fig 3(B)) shows the similarity of the microbial communities in various reaction stages [32]. Some 169 OTUs were unique to the A1-a sample and had no direct correlation with the rest of the reaction stages. The microbial communities of A1-a and A1-b were both from the anaerobic stage, which had the highest similarity, sharing 272 OTUs, while the microbial communities of A1-b and O had the weakest correlation, sharing only 172 OTUs. The results indicate that spatiotemporal distance affected the similarity of microbial communities, the greater the distance, the lower the similarity. The similarity of microbial communities between A1-a and O was due to the backflow of wastewater.

#### Composition of microbial communities

**Fig 4:**
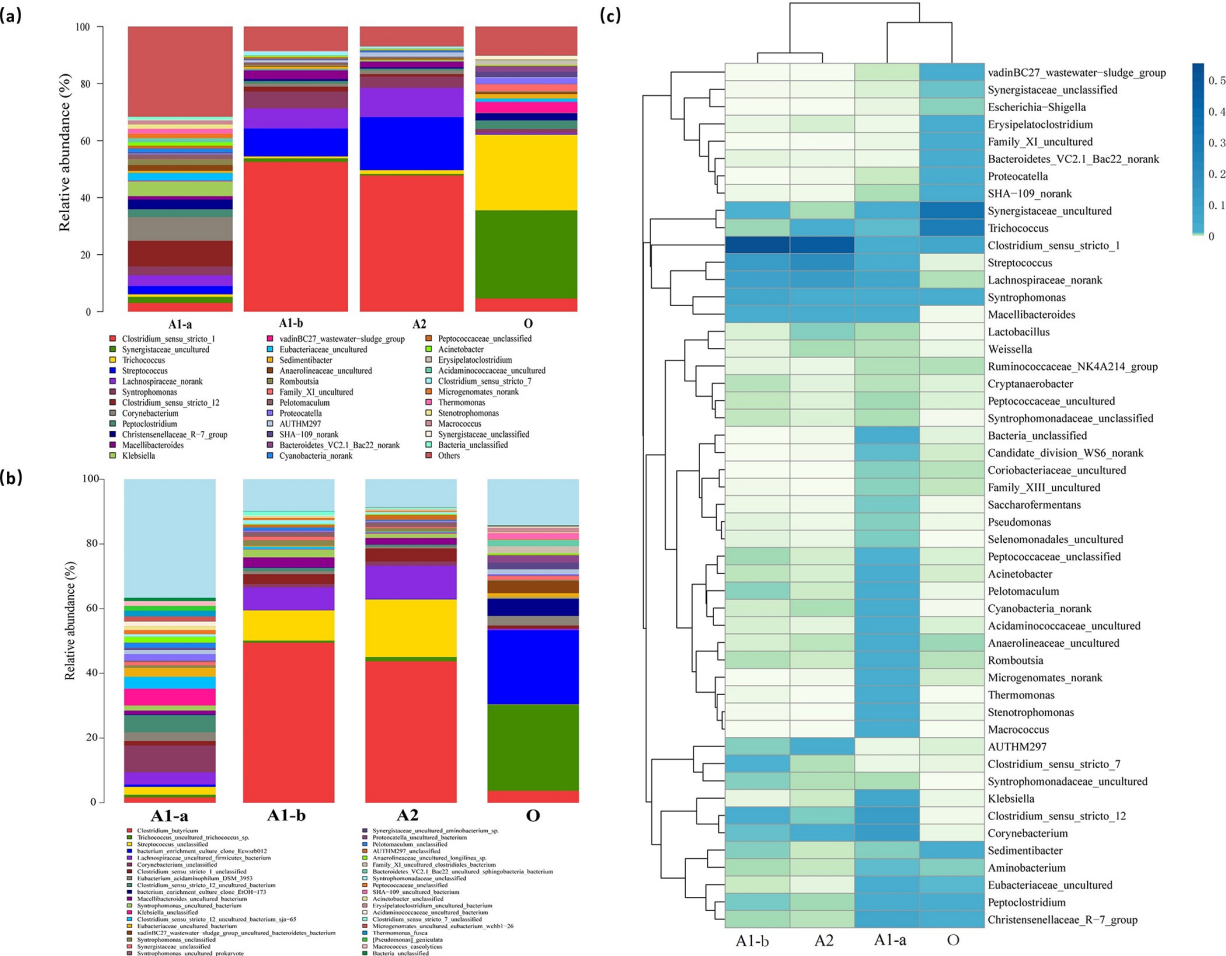
Microbial community composition. (a) distribution of genera; (b) distribution of species; and (c) heatmap of genera.

The genera of the microbial communities are shown in Fig 4. The difference in the abundance of genera between every biological phases can be directly observed. The microbial community structure showed that the SO reactor had the highest species richness and evenness, followed by the SR reactor. The SR reactor had high richness but low evenness, while the aerobic reactor had significantly lower richness and evenness than the SR and SO reactors, which was consistent with the conclusions in Table 3 and Fig 3(A).

With the enhanced tolerance and adaptability of microorganisms to wastewater pollutants, the genera richness of the microbial community was significantly decreased from anaerobic inlet section (A1-a) to anaerobic outlet section (A1-b). Through the competitive exclusion, dominant species with a high abundance, such as *Clostridium*, *Synergistaceae*, *Lachnospiraceae*, etc., appeared. However, the microbial community compositions of A1-b and A2 were similar, mainly including *Clostridium* and *Streptococcus*. While the aerobic reactor (O) was quite different, the main genera in it were *Trichococcus* and *Synergistaceae*.

#### Analyse the function of microbial communities by BESI^®^ process

The dominant genera of A1-a (the inlet section) showed in Fig 4(C) included *Corynebacterium* (8.31%), *Acinetobacter* [9] (1.15%) and *Pseudomonas* [33] (0.90%), which had been reported that they have Cr(VI) removal capability or participate in Cr(VI) removal [24, 34–37]. Among them, *Pseudomonas* had also been found to play a significant role in denitrification [38, 39]. Besides, the genus *Klebsiella* (5.18%) had been screened and isolated to remove the heavy metals Cu and Hg [40]. *Macellibacteroides* (1.18%) and *Trichococcus* (0.92%) could promote the reduction of sulfate and sulphite [41–43].

However, *Clostridium* (52.54%) was the most abundant genus of A1-b, which could desulfurize alkyl and aryl sulfonate [44], indirectly reducing organic sulfur compounds to HS^-^ [45]. Therefore, this genus may play a role in the release of sulfur from complex organics (such as aromatic hydrocarbons). Besides, it has been reported to be the functional bacteria of Cr removal [46]. The strictly anaerobic SRB including *Desulfobacteraceae*, *Desulfobulbus*, *Desulforhabdus*, *Desulfosporosinus* and *Desulfovibrio*, could be detected in the microbial community. The fermentative bacteria *Trichococcus* (0.66%) and *Macellibacteroides* (3.13%) can decompose complex organics into acetate, formate and lactate, and then promote the reduction of sulfate/sulphite, which have been reported that they can tolerate heavy metals such as lead and mercury for a long time [11, 43].

The abundant bacterial genera in the anoxic stage (A2) were similar to those of A1-b showed in Fig 4(C). The main taxa included *Clostridium* (47.78%), *Streptococcus* (18.58%), *Lachnospiraceae_norank* (10.27%), *Syntrophomonas* (3.98%), *Macellibacteroides* (2.04%), *AUTHM297* (1.54%), *Corynebacterium* (1.32%) and *Trichococcus* (1.31%). Compared to the SR reactor, the proportion of *Clostridium is* still high (47.78%), indicating that a large number of refractory and macromolecular organic pollutants have been further degraded in the SO reactor.

Besides, the microbial function of aerobic reactor (O) had changed greatly. The most abundant bacterial taxa were *Synergistaceae* (31.90%) and *Trichococcus* (26.59%). Among them, *Synergistaceae*, *Trichococcus*, *Clostridium sensu stricto 1*, and *vadinBC27* can decompose simple organics such as glucose into acids, CO_2_ and H_2_O [47–49], which were mostly small molecular organic-degrading bacteria. It showed that the function of microbial community has been transformed from macromolecular organic-degrading bacteria to small-molecular organic-degrading bacteria. Additionally, the BESI^®^ process achieved efficient removal of toxic chromium ions in the SR reactor and contributed to the subsequent gradient degradation of organic pollutants by functional microorganisms in SO and aerobic reactors. Functional degradation by microbial community was consistent with the results in Fig 2(E).

### Characterization of Cr-containing sludge

**Fig 5:**
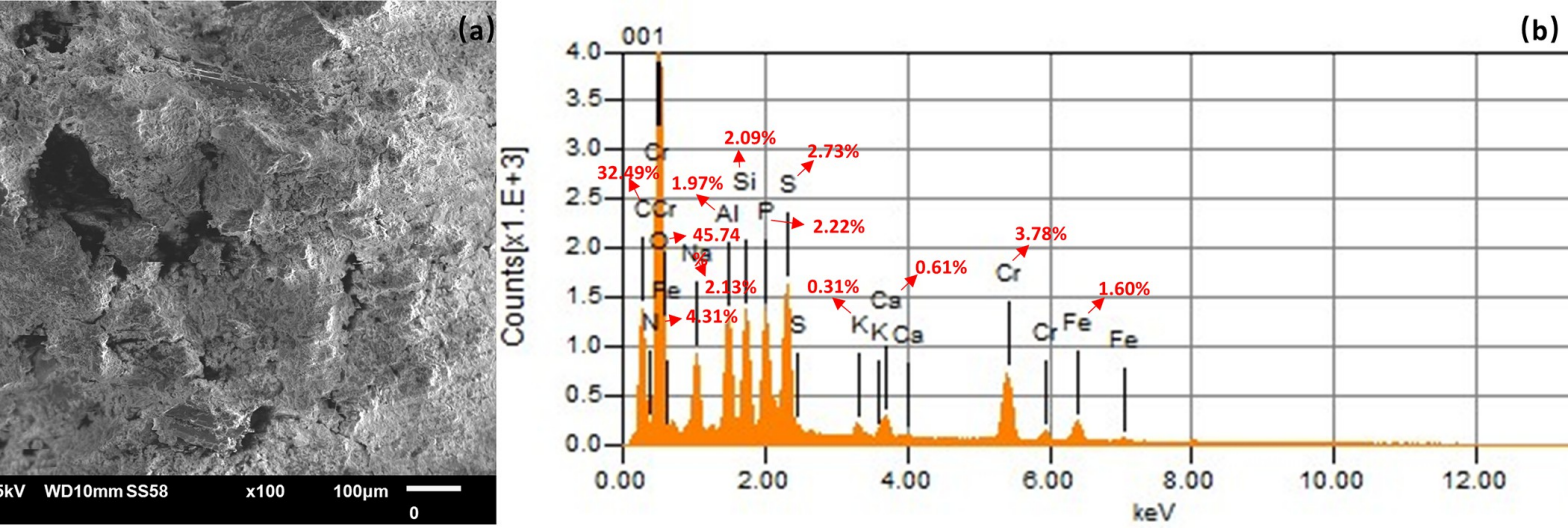
Analysis of Cr in sludge. (a) SEM image of sludge; (b) EDS analysis of sludge composition (Atomic percentage of the Chemical).

After 70 days of operation, grey-green sludge was found to adhere to the inner wall of the SR reactor. To confirm whether the grey-green precipitate was Cr(OH)_3_ precipitate, the sludge was collected from the SR reactor and dried to constant weight. SEM with EDS analysis was performed to test the composition of the sludge. The results are shown in Fig 5.

Fig 5(B) showed that the Cr content in the sludge was high. The atomic percentage of Cr reached 3.78%, accounting for 10.78% of the total mass of the sludge. The results indicated that the Cr ions in the wastewater had been transferred to sludge, achieving the goal of removing Cr ions from wastewater. Also, sulfur was high in the sludge, with an atomic percentage of 2.73%, speculating that sludge acclimated SRB or elemental sulfur was formed [11].

### The mechanism of Cr removal and biological detoxification

**Fig 6:**
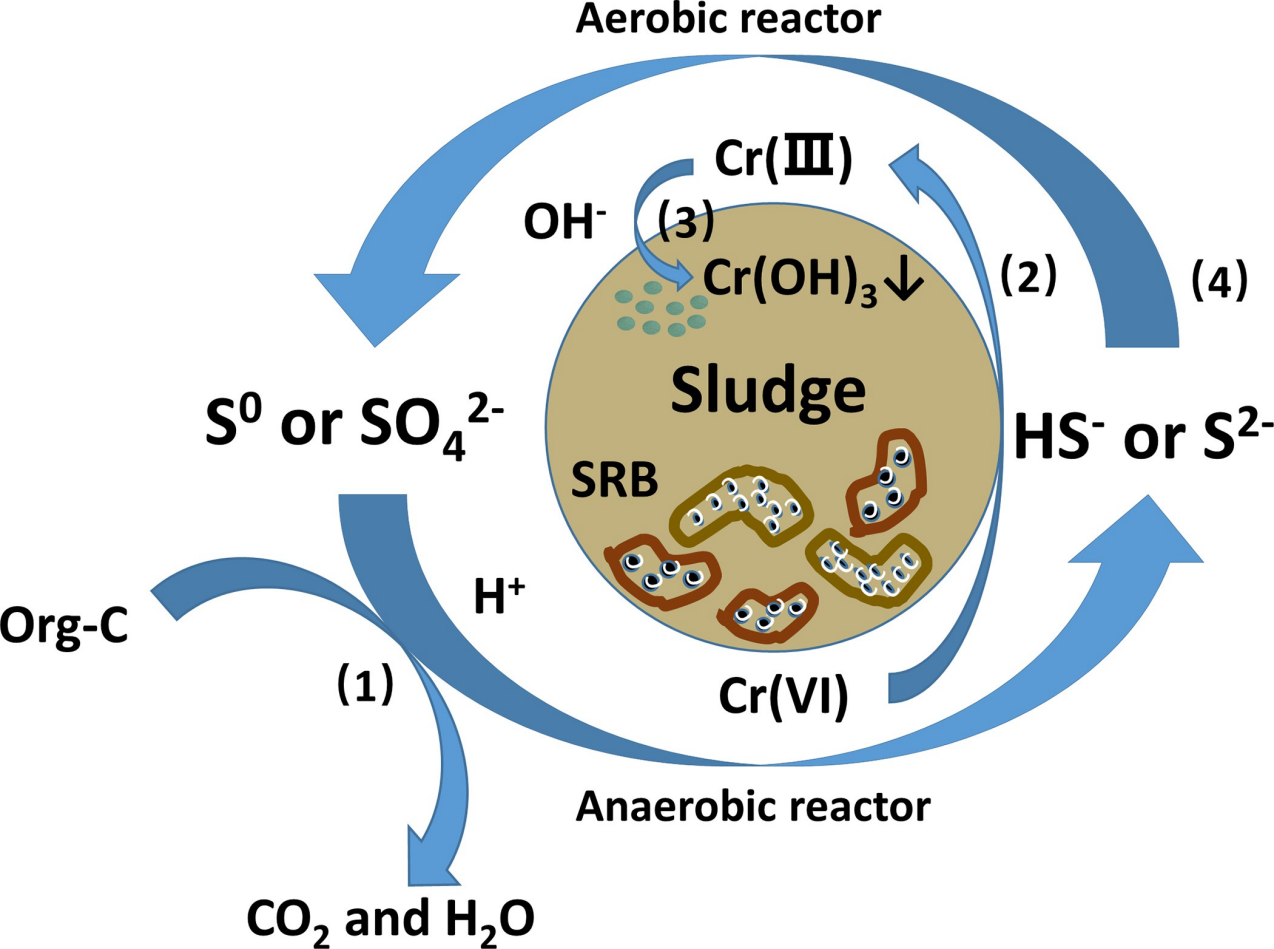
Cr removal mechanism by the BESI^®^ process.

Biological removal of Cr(VI) is achieved by the resistance of organisms to the toxicity of Cr(VI). The removal ways included biosorption, microbial uptake, microbial precipitation and enzymatic biotransformation [34, 50]. In this study, the reaction mechanism of Cr removal from wastewater by the BESI^®^ process can be speculated as follow (shown in Fig 6): (1) SRB in sludge used SO_4_^2-^ to produced sulfides from wastewater while both COD and H^+^ were consumed at the same time, as is shown in Eq (1) [4]. (2) S^2-^ reduced the more toxic Cr(VI) to less toxic Cr(Ⅲ) and generated S^0^ as shown in Eq (2), which H^+^ participated in this reaction [51]. However, for every 1 mol of Cr(VI) removed, 2 mol of H^+^ is consumed to produce S^0^. In this study, the increase in pH (from 2–3 to 6–8.5) is far from sufficient to reduce such a high concentration of Cr(VI) (initial concentration was 25–32 mg/L), so the gain and loss of H^+^ might be a cyclical process. (3) The increase in pH (pH>6.5) promoted the conversion from Cr(Ⅲ) to grey-green Cr(OH)_3_^0^ that precipitated in the sludge, thereby achieving the removal of Cr ions from wastewater. (4) The generation of S^0^ or SO_4_^2-^ produced by the reduction of Cr(VI) could be reused by SRB [45], which continued to promote the reduction of Cr(VI), when OH^-^ is produced in this process [52, 53]. Therefore, Cr(VI) can be completely removed from the wastewater through this cyclic conversion process.


$$1/2{{SO}_{4}}^{2-}+{CH}_{2}O+1/2H^{+}\to 1/2{HS}^{-}+H_{2}O+CO_{2}$$ (1)

$$2{{CrO}_{4}}^{2-}+3H_{2}S+4H^{+}\to 2{Cr(OH)}_{3(s)}+3{S^{0}}_{\left(s\right)}+2H_{2}O$$ (2)

However, the mechanism of biological detoxification from Cr manifested that the competitive inhibition between sulfate and Cr(VI) increased the tolerance threshold of bacteria to Cr(VI) in SR reactor. The production of sulfide reduced the more toxic Cr(VI) to less toxic Cr(Ⅲ), alleviating the toxic damage of synergistic bacteria in SR reactor, and contributing the sludge microbial communities degrade and remove other pollutants in the follow-up SO and aerobic reactors.

## Conclusions

The BESI® process effectively treated Cr-containing PCB wastewater in this study. The values of total Cr, Cr(VI) and pH of the effluent met the wastewater discharge standards for the electroplating industry. Furthermore, the COD removal efficiency reached 80% even though the influent COD was as high as 1000 mg/L. OTUs results showed that the SR reactor exhibited the highest microbial community diversity (A1-a>A1-b>A2>O), enabling microorganisms to withstand the impact of wastewater containing high concentrations of COD and toxic Cr ions. After analyzing the microbial community, *Clostridium* (52.54% and 47.78%) was the dominant bacteria in both the SR and SO reactor contributing to the reductions in organic sulfur compounds in wastewater. While the *Synergistaceae* (31.90%) and *Trichococcus* (26.59%) were predominant in aerobic reactor. The removal of Cr from wastewater was significantly correlated with sulfide generation in the SR reactor (R^2^ = 0.9987). EDS detected Cr, indicating that Cr(VI) from wastewater had been transformed into grey-green Cr(OH)_3_ precipitates in sludge through the involvement of sulfide and H^+^ as electron donors. The BESI^®^ process demonstrated high efficiency and stability in treating wastewater containing Cr from PCBs. These findings may serve as a foundation for the development of biodegradation methods to treat electroplating industry wastewater.
